# An Optimized Method for Quantification of Pathogenic *Leptospira* in Environmental Water Samples

**DOI:** 10.1371/journal.pone.0160523

**Published:** 2016-08-03

**Authors:** Irina N. Riediger, Alex R. Hoffmaster, Arnau Casanovas-Massana, Alexander W. Biondo, Albert I. Ko, Robyn A. Stoddard

**Affiliations:** 1 Central Laboratory of the State of Paraná, Curitiba, Paraná, Brazil; 2 National Center for Emerging and Zoonotic Infectious Diseases, Centers for Disease Control and Prevention, Atlanta, Georgia, United States of America; 3 Department of Epidemiology of Microbial Diseases, School of Public Health, Yale University, New Haven, Connecticut, United States of America; 4 Department of Veterinary Medicine, Federal University of the State of Paraná, Curitiba, Paraná, Brazil; 5 Gonçalo Moniz Research Center, Oswaldo Cruz Foundation, Brazilian Ministry of Health, Salvador, Bahia, Brazil; University of Kentucky College of Medicine, UNITED STATES

## Abstract

Leptospirosis is a zoonotic disease usually acquired by contact with water contaminated with urine of infected animals. However, few molecular methods have been used to monitor or quantify pathogenic *Leptospira* in environmental water samples. Here we optimized a DNA extraction method for the quantification of leptospires using a previously described Taqman-based qPCR method targeting *lipL32*, a gene unique to and highly conserved in pathogenic *Leptospira*. QIAamp DNA mini, MO BIO PowerWater DNA and PowerSoil DNA Isolation kits were evaluated to extract DNA from sewage, pond, river and ultrapure water samples spiked with leptospires. Performance of each kit varied with sample type. Sample processing methods were further evaluated and optimized using the PowerSoil DNA kit due to its performance on turbid water samples and reproducibility. Centrifugation speeds, water volumes and use of *Escherichia coli* as a carrier were compared to improve DNA recovery. All matrices showed a strong linearity in a range of concentrations from 10^6^ to 10° leptospires/mL and lower limits of detection ranging from <1 cell /ml for river water to 36 cells/mL for ultrapure water with *E*. *coli* as a carrier. In conclusion, we optimized a method to quantify pathogenic *Leptospira* in environmental waters (river, pond and sewage) which consists of the concentration of 40 mL samples by centrifugation at 15,000×g for 20 minutes at 4°C, followed by DNA extraction with the PowerSoil DNA Isolation kit. Although the method described herein needs to be validated in environmental studies, it potentially provides the opportunity for effective, timely and sensitive assessment of environmental leptospiral burden.

## Introduction

Leptospirosis is a zoonotic disease caused by pathogenic spirochetes of the genus *Leptospira* [1,2]. Clinical symptoms range from mild flu-like infections to life-threatening manifestations such as Weil’s disease and pulmonary hemorrhage, the latter showing case fatality rates as high as 50% [3,4]. Leptospirosis has become particularly prevalent in poor urban and peri-urban communities from tropical developing countries [5–7]. In such locations, deficient sewer, drainage and refuse collection systems provide optimal ecological conditions for the thriving of synanthropic rodents such as *Rattus rattus* and *R*. *norvergicus*, which are natural reservoirs of pathogenic leptospires [8,9]. Human infection occurs predominantly by contact of abrasions or cuts in the skin or mucous membranes with water or soil contaminated with urine of rodents chronically infected with leptospires [2,10,11]. Large leptospirosis outbreaks are commonly associated to heavy rainfall and flooding events [12–17] and outdoor recreational activities involving water contact [18–22], highlighting its waterborne transmission.

Transmission dynamics and clinical progression are modulated by virulence characteristics of the infecting strain, by the host immune status and inherent susceptibility factors; and by the *Leptospira* inoculum size [2,23]. Thereby, the assessment and quantification of the leptospiral burden in environmental water samples would be valuable to guide public health interventions, and help expand the current knowledge about the leptospirosis zoonotic cycle and the spatial and temporal distribution of leptospires in the environment. Specifically, the availability of quantitative methods would allow the evaluation of the impact of interventions through the observation of decreasing environmental bacterial loads. Furthermore, the determination of the environmental burden may help to inform health authorities before adopting preventive measures such as closing recreational areas or access to rivers or other water sources during heavy rainfall events.

To date, culture isolation and animal inoculation have been the most used methods to detect leptospires in the environment. These methods are laborious and time-consuming and their lack of sensitivity may lead to false-negative results [24]. Inhibitors potentially present in complex environmental samples can hinder *in vitro* growth of leptospires and impair their isolation. Additionally, pathogenic leptospires are easily outgrown in culture by contaminating bacteria and saprophytic leptospires [1,11]. The use of molecular methods might overcome the limitations inherent to culture- and animal-based methods and provide quantitative information about the concentration of leptospires in contaminated water. A number of studies have successfully quantified pathogenic *Leptospira* in environmental waters (i.e. wells, streams, rivers, spring and standing water) using qPCR assays targeting the 16S rRNA [23,25] or *lipL32* genes [26–28].

Different centrifugation or filtration methods and commercial DNA extraction kits were used, but only Vein et al., 2012 [27] compared the efficiency of the DNA extraction procedures. However, the comparison was based exclusively on the total quantity of DNA extracted and the linearity and lower limit of detection were not determined. In this study we aimed to optimize a method to concentrate and extract leptospiral DNA from environmental water samples to be quantified with a previously described Taqman-based qPCR method targeting the *lipL32* gene of pathogenic *Leptospira* species [29]. This qPCR has been successfully applied in clinical samples [15] and animal tissues [30–32] showing high specificities. This procedure provides the opportunity for an effective and timely assessment of environmental leptospiral risk.

## Materials and Methods

### Water samples

Three water matrices were collected within the metropolitan area of Atlanta, GA, USA in March, 2010 and tested in this study. Raw sewage was provided by the staff of Snapfinger Creek Advanced Wastewater Treatment Facility (Decatur, GA, USA). Pond water was collected with the permission of the land owner from a pond located in Decatur, GA (N33°48’07” W84°18’26”) and river shoreline water was collected in the Chattahoochee River National Recreation Area in Roswell, GA (N33°59’17” W84°17’38”), with the permission of the Park Research Coordinator. The water collection procedures did not involve or affect any endangered and protected species. In all cases, an eight-liter batch was collected in a clean container and stored at 4°C until use. Ultrapure, distilled, nuclease-free water (Gibco BRL, Gaithersburg, USA) was used in all the experiments as an inhibitor-free control.

### Bacterial strains and culture

*L*. *interrogans* serovar Copenhageni strain Fiocruz L1-130 (ATCC^®^ BAA-1198) and *Escherichia coli* strain F2747 (provided by the Special Bacteriology Reference Laboratory, CDC) were used in the spiking experiments. *L*. *interrogans* was grown in liquid Ellinghausen-McCullough-Johnson-Harris (EMJH) [33,34] medium at 29°C for five days and counted using a Petroff-Hauser chamber under dark-field microscopy. *E*. *coli* was cultured in blood agar plates at 37°C for 24 hours and the concentration was estimated by suspending isolated colonies in sterile PBS (0.01 M, pH = 7.2) and correcting the density to 0.5 McFarland’s scale.

### Selection of DNA extraction kit

Four aliquots of 40 mL of each water matrix were spiked with 1 × 10^5^ leptospires/mL and centrifuged at 3,000 × *g* for 20 minutes at room temperature. The resuspended pellets were submitted to DNA extraction using either the QIAamp DNA mini kit (QIAGEN, Valencia, CA) or the PowerSoil DNA Isolation kit (MO BIO, Carlsbad, CA), following the manufacturers’ instructions. PowerWater DNA Isolation kit (MO BIO, Carlsbad, CA) was used to extract DNA from two additional, non-centrifuged aliquots of each water matrix. Samples were filtered through 11μm filter papers to remove large particulate debris and filtrates were passed through 0.22 μm membranes, which were aseptically removed and used for DNA extraction as per manufacturer’s recommendation. For consistency, the experiment was repeated three independent times in duplicates. For further experiments, DNA extraction was performed using PowerSoil DNA Isolation kit (MO BIO).

### Optimization of the concentration protocol

To evaluate the efficiency of two centrifugation protocols, aliquots of 40 mL of each water matrix were spiked with 1 × 10^5^ leptospires/mL and centrifuged using either protocol A (3,000 × *g* for 20 minutes at room temperature), or protocol B (15,000 × *g* for 20 minutes at 4°C). After careful removal of supernatants, the resuspended pellets were submitted to DNA extraction. As a control for loss of deposited matter due to supernatant removal, DNA was extracted from 200 μl of each spiked sample without a previous centrifugation step. To improve cell pelleting, 40 mL aliquots of ultrapure water spiked with 1 × 10^5^ leptospires/mL were additionally spiked with varying amounts of *E*. *coli* (none, 1 × 10^5^, 1 × 10^6^ and 1 × 10^7^/mL) as a carrier [35]. An ultrapure water sample spiked only with 1 × 10^7^/mL *E*. *coli* was used as a negative control. All experiments were repeated three independent times in duplicates. Subsequent experiments were performed using the centrifugation procedure B.

### Selection of the optimal sample volume

To determine the optimal sample volume, aliquots of 40, 80, 200 and 400 mL of each water matrix were spiked with 1 × 10^5^ leptospires/mL and 1 × 10^7^ *E*. *coli*/mL (except for sewage), centrifuged using the centrifugation procedure B, and pellets were submitted to DNA extraction. The experiment was repeated three independent times in duplicates. Subsequent experiments were conducted using 40 mL of water.

### Linearity of extraction and determination of lower limit of detection (LLOD)

To compare the extraction linearity over a range of concentrations and determine the LLOD for each water matrix, 40 mL of each sample were spiked with a range of concentrations from 1 × 10^6^ leptospires/mL to 1 × 10° leptospires/mL. All samples were also spiked with 1 × 10^7^ *E*. *coli*/mL, with the exception of sewage. For comparison purposes, an additional group of ultrapure water samples was spiked with *Leptospira* but no *E*. *coli*. All samples were centrifuged and submitted to DNA extraction as described above. All experiments were repeated three independent times in duplicates.

### Quantitative real-time PCR

DNA extracts were tested in triplicate by qPCR using previously described oligonucleotides [29]. The reaction mix consisted of 12.5 μl of Platinum Quantitative PCR SuperMix-UDG (Invitrogen, Carlsbad, CA), 500 nM of forward and reverse primers, 100 nM of probe, 5 μl of DNA extract and ultrapure water to a final volume of 25 μl. Amplification was performed on a ABI 7500 Real-Time PCR System (Applied Biosystems, Foster, CA) using standard conditions for 45 cycles. All samples with quantification cycles lower than 40 were considered positive. Genomic DNA obtained from *L*. *interrogans* serovar Copenhageni strain Fiocruz L1-130 was used to construct a standard curve with concentrations ranging from 1 × 10^7^ to 1 × 10° GEq/5 μl, based on its genome size of 4,627 Mb [36]. Non-template controls were included after every five samples. All water matrices were proven negative for the presence of pathogenic *Leptospira* by qPCR before being used in the experiments. No amplification signal was detected when water samples spiked exclusively with *E*. *coli* were tested by qPCR.

### Statistical analysis

To facilitate the comparison among experiments performed with different sample volumes, the recovery of leptospiral DNA was expressed in GEq/mL in all cases. All results were log_10_-transformed prior to further statistical analysis. Student’s unpaired *t* test was used to assess the difference between the means when two groups were compared and Tukey-Kramer *post hoc* test was used to correct for multiple comparisons. A linear regression model was used to evaluate the DNA extraction linearity over a range of concentrations and the slopes and y-intercepts of best-fit lines were compared by analysis of covariance. Probit regression analysis was used to estimate the LLOD targeting a 95% hit-rate (IBM SPSS software v.19, SPSS Inc., Chicago, IL). All the other statistical analysis were performed using GraphPad Prism 6.02 (GraphPad Software, San Diego California).

## Results and Discussion

In this study we have optimized a method for the sample concentration and DNA extraction of environmental water matrices (river water, pond water and sewage) that may be used to monitor the leptospiral burden using a previously described qPCR method [29]. First, we evaluated the performance of three commercial kits: PowerWater DNA Isolation kit (MO BIO), QIAamp DNA Mini kit (QIAGEN) and PowerSoil DNA Isolation kit (MO BIO). PowerWater kit recovered more DNA than the others for river and ultrapure water samples (Table 1), but PowerSoil kit showed the highest efficiency when DNA was extracted from turbid samples (sewage and pond water) (p<0.0001). In addition, it presented the lowest DNA recovery variability among the four water types, being thus selected for further experiments. The underperformance of PowerWater kit for pond and sewage samples might be due to the clogging of the filter at the filtration step, which limits its ability to process large volumes of water. Furthermore, previous studies have reported the outperformance of MO BIO DNA extraction kits over QIAamp DNA mini kit for particulate-rich water samples [37–39]. The two MO BIO kits, specifically optimized to extract DNA from environmental samples, include a bead beating step that improves both bacterial desorption from sediments and cell disruption. DNA shearing caused by the bead beating process may lead to weaker interactions with sample particles than those that would be observed with intact DNA. Shorter, fragmented DNA would therefore be easier to desorb during DNA extraction [40]. In addition, MO BIO kits include a step for cationic flocculation of humic substances, which are removed prior to column loading. As a result, these chemical complexes do not compete with DNA for the silica binding sites in the column, leading to a higher final DNA recovery [41].

**Table 1:** Comparison of the efficiency of DNA extraction for three commercial kits from four water types spiked at 1 × 10^5^ *Leptospira*/mL.

	mean log_10_ *Leptospira* GEq/mL ± SD^†^		
Water matrix	PowerWater DNA Isolation Kit^‡^	QIAamp DNA Mini Kit^§^	PowerSoil DNA Isolation Kit^§^
Ultrapure	4.62 ± 0.30	3.27 ± 0.15	3.33 ± 0.02
River	4.65 ± 0.01	4.56 ± 0.09	3.71 ± 0.18
Pond	3.91 ± 0.13	Not detected	4.17 ± 0.09
Sewage	1.90 ± 0.62	0.68 ± 0.59	3.37 ± 0.02

^†^Data represent the mean results obtained from three independent experiments. Statistical analysis revealed that the difference in DNA extraction efficiency upon comparison of the three kits tested was significant and was observed for all the four water matrices (*P*<0.0001).

^‡^DNA was extracted from 40 mL aliquots, without a centrifugation step.

^§^Forty mL aliquots were centrifuged at 3,000×g for 20 minutes at room temperature. The respective pellets were used for DNA extraction.

**Fig 1:**
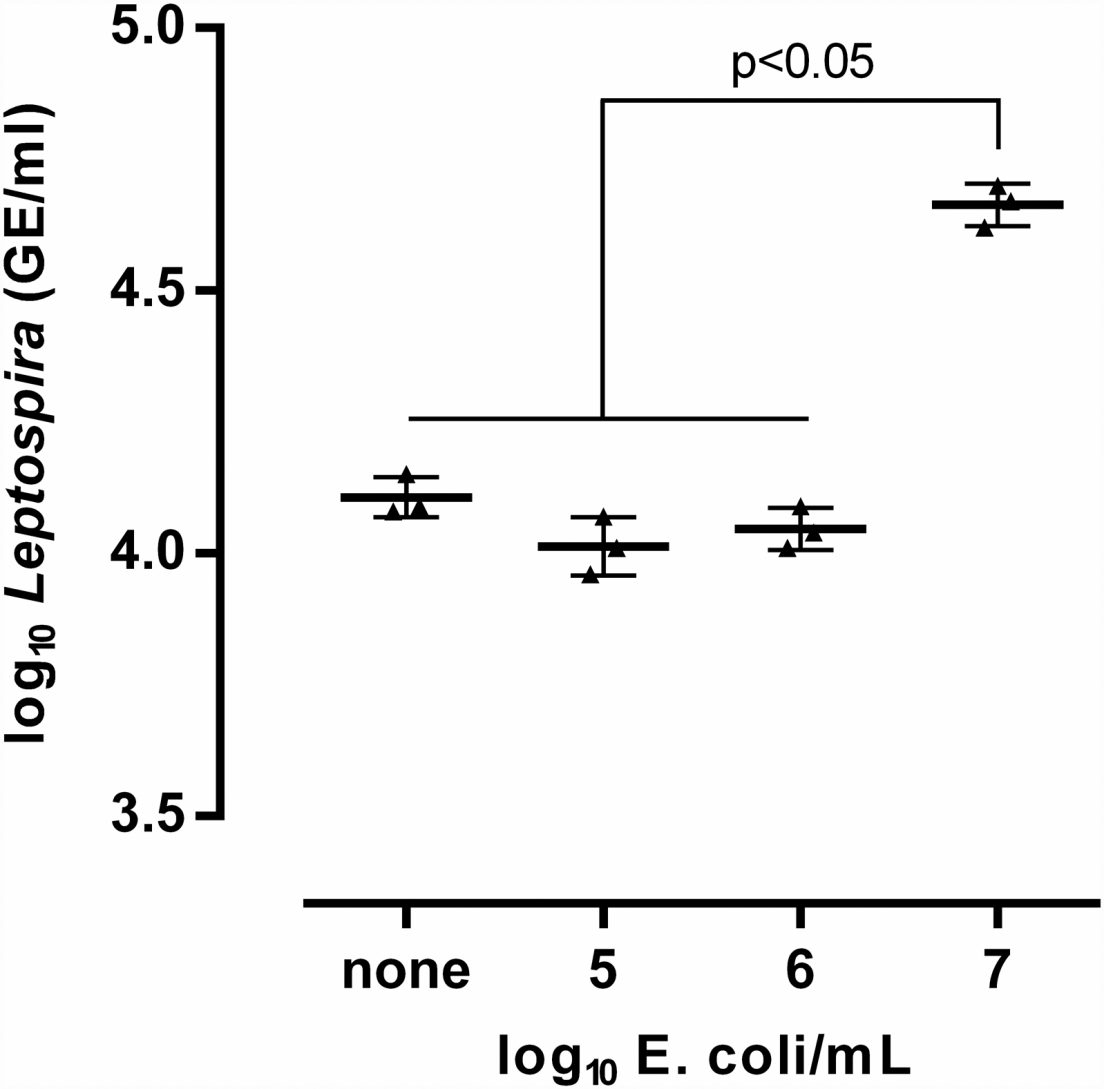
Effect of the addition of varying amounts of *E*. *coli* on *Leptospira* recovery from ultrapure water. Aliquots were spiked with 1 × 10^5^ *Leptospira*/mL and with 1 × 10^5^, 1 × 10^6^ and 1 × 10^7^ *E*. *coli*/mL. Ultrapure water spiked with 1 × 10^5^ *Leptospira*/mL was used as negative control. Error bars represent the geometric mean ± SD of the concentrations as determined by qPCR in three independent experiments.

Two different centrifugation protocols (A: 3,000 ×*g* for 20 minutes at room temperature, and B: 15,000×g for 20 minutes at 4°C) were compared using the MO BIO PowerSoil DNA Isolation kit. These two centrifugation speeds have been used in previous studies [23,27,26], although the specific effect of the speed in the recovery of DNA has not been determined. The difference between mean leptospiral DNA quantities obtained with both centrifugation protocols was not statistically significant, except for river water in which more DNA was recovered using protocol B (p = 0.0005). Both protocols yielded lower recoveries than those obtained from the aliquots which were not centrifuged (p < 0.05) (Table 2). Despite these lower recoveries, the centrifugation protocol B was chosen for further tests because it allowed the concentration of at least 200 times more sample volume than the direct extraction (40 ml and 200 μL, respectively). Since the concentration of pathogenic leptospires in environmental water is presumably low (~10^3^ leptospires/ml even in high-risk endemic areas [23]), larger volumes are required to provide adequate load estimations. In parallel, we also evaluated whether the addition of different amounts of *E*. *coli* as a carrier would improve leptospiral DNA recovery from ultrapure water. The addition of 1 × 10^7^ *E*. *coli*/mL to ultrapure water significantly improved the recovery of leptospiral DNA (p < 0.05), but had no effect with smaller concentrations (Fig 1). Thus, *E*. *coli* can act as a carrier in clear water and co-sediment with leptospires to generate larger and more visible pellets less prone to be washed away upon supernatant removal. Consequently, 1 × 10^7^ *E*. *coli*/mL was added to water aliquots used in the subsequent tests, except for sewage. We did not add *E*. *coli* to sewage because it naturally contains large amounts *E*. *coli* and other enteric bacteria, which already produce large visible pellets.

**Table 2:** Effect of different centrifugation protocols on the recovery of leptospiral DNA from four water samples spiked at 1 × 10^5^ *Leptospira*/mL.

	mean log_10_ *Leptospira* GEq/mL ± SD			
Water matrix	No centrifugation^†^	Centrifugation Protocol		
		A^‡^	B^§^	*P* value
Ultrapure	4.92±0.15	3.34±0.17	3.42±0.09	0.5360
River	4.39±0.02	3.35±0.01	3.77±0.07	0.0005
Pond	4.50±0.01	4.20±0.09	4.18±0.08	0.7950
Sewage	4.25±0.12	3.58±0.07	3.44±0.05	0.0512

^†^DNA was extracted from 200μL aliquots, without a centrifugation step.

^‡^Forty mL aliquots were centrifuged at 3,000×g for 20 minutes at room temperature. The respective pellets were used for DNA extraction.

^§^ Forty mL aliquots were centrifuged at 15,000×g for 20 minutes at 4°C. The respective pellets were used for DNA extraction

**Fig 2:**
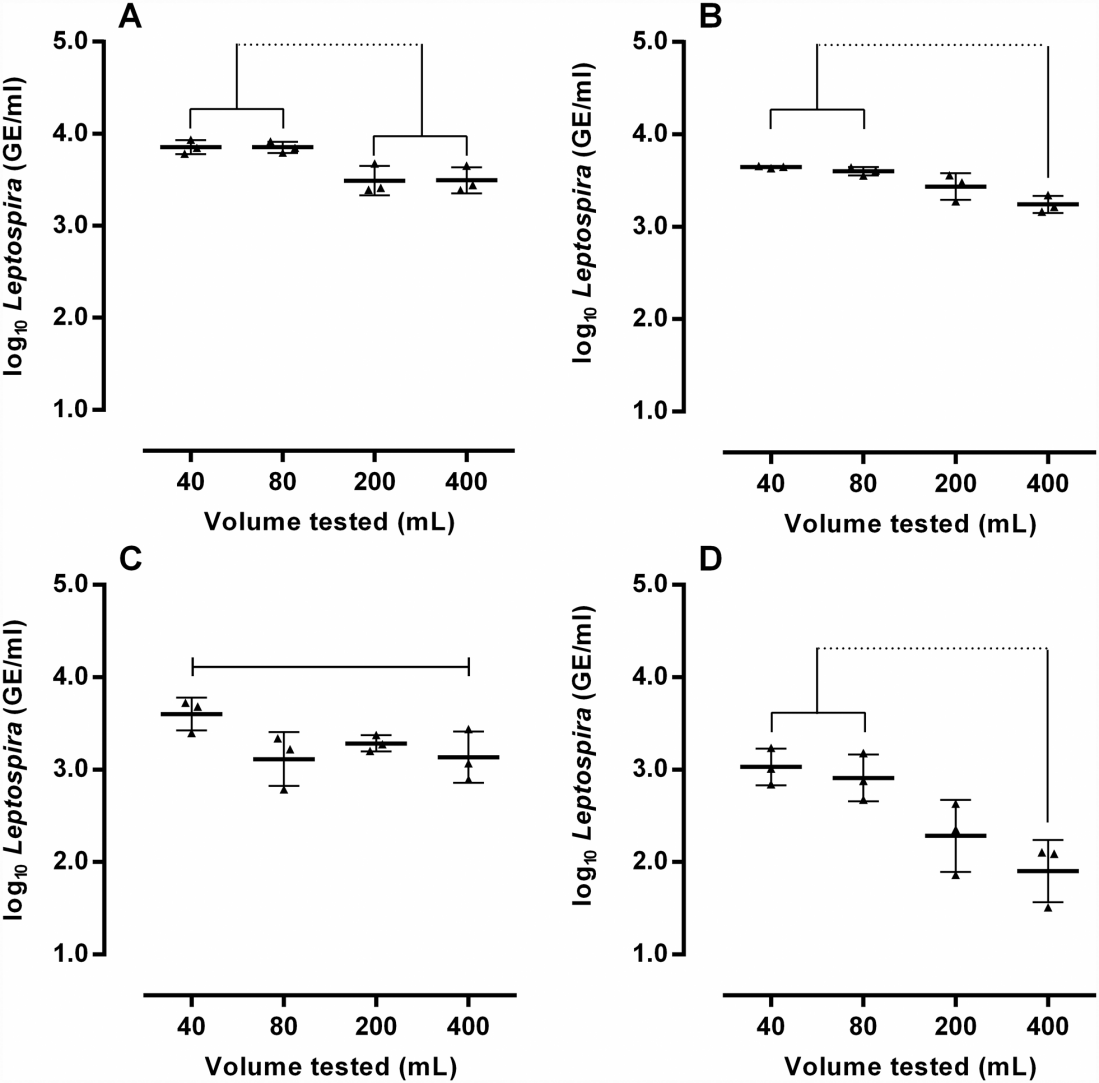
Determination of the optimal volume to be tested by qPCR for different water types: ultrapure water (A), river (B); pond (C) and sewage (D). Different volumes of each water type were spiked with 1 × 10^5^ leptospires/mL to assess *Leptospira* recovery. Error bars represent the geometric mean ± SD of the concentrations as determined by qPCR in three independent experiments. Continuous lines connect groups whose average leptospiral DNA concentrations are not statistically significant (p>0.05). Groups whose averages are significantly different (p<0.05) are connected by a dashed line.

We then compared the efficiency of DNA extraction using different sample volumes (40, 80, 200 and 400 mL) to determine the best initial volume for *Leptospira* quantification in environmental waters. For all samples except pond water, qPCR quantification from 40 mL and 80 mL was not statistically different (Fig 2). However, leptospiral quantification obtained from 40 mL aliquots was significantly higher than those obtained from 200 mL and 400 mL aliquots for the four samples. For all the water matrices, an increase in sample volume led to a decrease in leptospiral DNA quantification (Fig 2). This observation may be due to a higher loss of bacteria during centrifugation with larger sample volumes or a saturation of the binding capacity of the DNA extraction kit. Alternatively, although the PowerSoil DNA Isolation kit had been designed to remove inhibitory substances from environmental samples, we cannot rule out the possibility that the amount of inhibitors contained in large water aliquots surpassed the kit’s removal capacity, particularly in sewage. A limitation of our study is the lack of an internal control to monitor the efficiency of DNA extraction and PCR amplification. Since it is not possible to precisely predict the amount of inhibiting compounds in environmental samples, an internal control would be useful to correlate the presence of inhibitors with DNA amplification efficiency [37].

**Fig 3:**
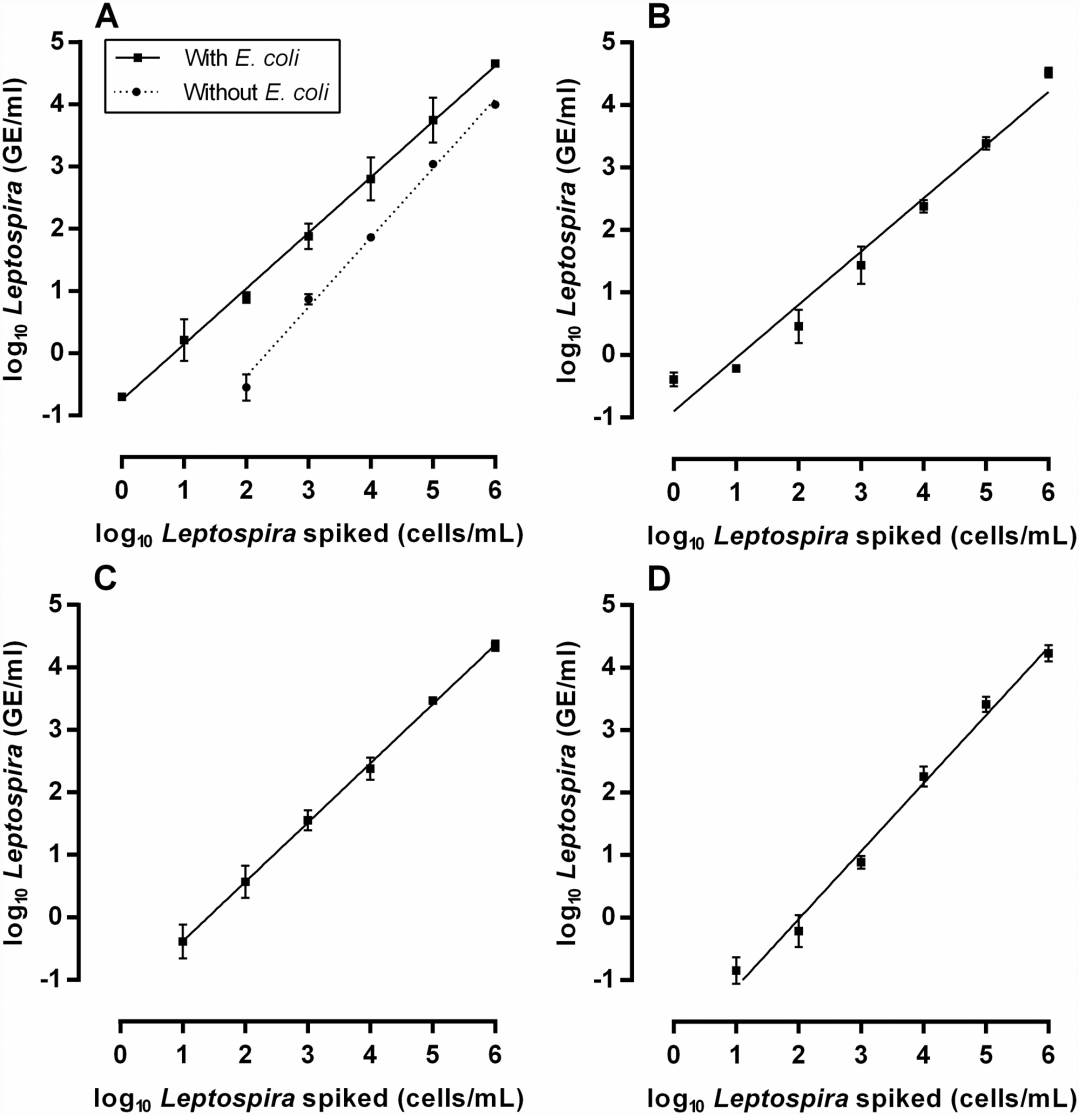
Determination of the lower limit of detection for water samples obtained ultrapure water (A), river (B), pond (C) and sewage (D). Ultrapure water (A) was tested with and without the addition of 1 × 10^7^ *E*. *coli*/mL. All the samples were spiked with 10^6^ *Leptospira*/mL and 10-fold serially diluted down to 1 *Leptospira*/mL. Error bars represent the geometric mean ± SD of three independent experiments.

Finally, we determined the range of linearity and the LLOD of the method by spiking 40mL of each water matrix with 1 × 10^6^ leptospires/mL to 1 × 10° leptospires/mL. As established in the previous experiment, 1 × 10^7^ *E*. *coli*/mL were added to all samples, except for sewage. We observed a strong linear relationship between the concentration of *Leptospira* spiked and the concentration determined by qPCR after the extraction procedure (R^2^ > 0.97 in all cases) (Table 3 and Fig 3). Analysis of covariance of the slope and y-intercept of the best-fit lines calculated for each of the five samples tested showed that all the lines were significantly different between them (p < 0.0001). The 95% hit-rate LLODs were estimated at 36 cells/mL for ultrapure water with *E*. *coli*, 4,833 cells/mL for ultrapure water without *E*. *coli*, 11 cells/mL for pond water and 18 cells/mL for sewage water. It was not possible to calculate the 95% hit-rate LLOD for river water since all the replicates tested were positive, which indicated that it was lower than 1 cell/mL. Altogether, the strong linearity and low LLOD in all the water matrices indicate that the optimized DNA extraction method is suitable for the detection of a wide range of *Leptospira* concentrations in environmental waters.

**Table 3:** Lower limit of detection and best-fit lines calculated from four water types spiked with leptospires*.

		Best-fit line^†^		
Water matrix	LLOD (cells/mL)^‡^	R^2^	Slope ± SD	Y-intercept ± SD
Ultrapure (with *E*. *coli)*	36	0.98	0.89 ± 0.027	-0.75 ± 0.101
Ultrapure (without *E*. *coli)*	4,833	0.99	1.11 ± 0.026	-2.60 ± 0.114
River	<1	0.97	0.85 ± 0.036	-0.90 ± 0.131
Pond	11	0.99	0.95 ± 0.023	-1.33 ± 0.091
Sewage	18	0.99	1.09 ± 0.034	-2.20 ± 0.137

* In three different experiments, water samples were spiked with 1 × 10^6^ *Leptospira*/mL and 10-fold serially diluted down to 1 × 10° *Leptospira*/mL

^†^Calculated from linear regression of data obtained from 3 independent experiments.

^‡^As determined by Probit regression analysis.

In summary, we optimized a method to quantify pathogenic *Leptospira* in environmental waters (river, pond and sewage). The process includes concentration of 40 mL samples by centrifugation at 15,000×g for 20 minutes at 4°C, followed by extraction with the PowerSoil DNA Isolation kit (MO BIO). Although the optimized method needs to be further validated in environmental studies, it is a promising tool for the environmental monitoring of pathogenic *Leptospira* that may help to inform public health interventions aimed to reduce the burden of the disease.
